# The role of intercostal nerve preservation in acute pain control after thoracotomy*

**DOI:** 10.1590/S1806-37132014000200010

**Published:** 2014

**Authors:** Marco Aurélio Marchetti-Filho, Luiz Eduardo Villaça Leão, Altair da Silva Costa-Junior

**Affiliations:** Department of Thoracic Surgery, Federal University of São Paulo/Paulista School of Medicine, São Paulo, Brazil; Department of Thoracic Surgery, Federal University of São Paulo/Paulista School of Medicine, São Paulo, Brazil; Department of Thoracic Surgery, Federal University of São Paulo/Paulista School of Medicine, São Paulo, Brazil

**Keywords:** Pain, postoperative, Analgesia, Thoracotomy

## Abstract

**OBJECTIVE:**

To evaluate whether the acute pain experienced during in-hospital recovery from thoracotomy can be effectively reduced by the use of intraoperative measures (dissection of the neurovascular bundle prior to the positioning of the Finochietto retractor and preservation of the intercostal nerve during closure).

**METHODS:**

We selected 40 patients who were candidates for elective thoracotomy in the Thoracic Surgery Department of the Federal University of São Paulo/Paulista School of Medicine, in the city of São Paulo, Brazil. The patients were randomized into two groups: conventional thoracotomy (CT, n = 20) and neurovascular bundle preservation (NBP, n = 20). All of the patients underwent thoracic epidural anesthesia and muscle-sparing thoracotomy. Pain intensity was assessed with a visual analog scale on postoperative days 1, 3, and 5, as well as by monitoring patient requests for/consumption of analgesics.

**RESULTS:**

On postoperative day 5, the self-reported pain intensity was significantly lower in the NBP group than in the CT group (visual analog scale score, 1.50 vs. 3.29; p = 0.04). No significant differences were found between the groups regarding the number of requests for/consumption of analgesics.

**CONCLUSIONS:**

In patients undergoing thoracotomy, protecting the neurovascular bundle prior to positioning the retractor and preserving the intercostal nerve during closure can minimize pain during in-hospital recovery.

## Introduction

A thoracotomy is one of the most painful procedures in surgical practice. The pain that patients experience in the immediate postoperative period and in the late postoperative period constitutes a constant concern among thoracic surgeons, because it is well established that patients with severe postoperative pain are at an increased risk of developing complications, including atelectasis and pulmonary infection.^(^ ^1^ ^,^ ^2^ ^)^ In addition, chronic pain is a common cause of prolonged work absenteeism because it often prevents patients from performing their regular activities for months after the surgical procedure. Many studies have shown that the presence of severe pain in the immediate postoperative period is associated with a higher occurrence of chronic pain.^(^ ^3^ ^-^ ^5^ ^)^

Postoperative pain assessment is based on individual perception, the subjective nature of pain and the difficulty in measuring pain intensity making it difficult to standardize studies addressing the issue of postoperative pain.

Pain resulting from the stimulation of receptors is designated nociceptive (myofascial) pain. Nociceptive pain after thoracotomy might be due to any of the following: skin incision; muscle retraction; rib spreading; trauma to sternocostal and costovertebral joints; intercostal nerve compression; damage to the lung parenchyma; and damage to the parietal pleura. Intercostal nerve injury can lead to the formation of a localized neuroma that can cause persistent stimulation and, consequently, hyperalgesia (pain resulting from noxious stimuli) and allodynia (pain resulting from typically painless stimuli). In such cases, pain is designated neuropathic pain. Post-thoracotomy pain syndrome can therefore be defined as a combination of nociceptive and neuropathic stimuli.^(^ ^6^ ^,^ ^7^ ^)^

Many thoracic surgical procedures are currently performed as video-assisted thoracoscopic procedures; however, pulmonary resections, including those for the treatment of lung cancer, are not, despite numerous studies showing the advantages of video-assisted thoracoscopy. According to the American Association for Thoracic Surgery, video-assisted procedures account for less than 20% of all major pulmonary resections recently performed in the USA and less than 10% of all major pulmonary resections recently performed in Europe.^(^ ^8^ ^)^

During conventional thoracotomy, two intercostal nerves can be injured unless the neurovascular bundle is preserved: one by the Finochietto retractor and one by thoracotomy closure. On the basis of the assumption that post-thoracotomy pain is primarily due to intercostal nerve injury, postoperative pain can be reduced by avoiding intercostal nerve crushing.

**Figure 1:**
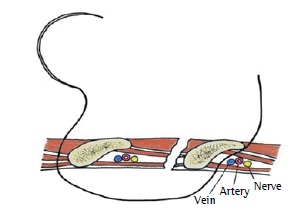
Schematic illustration of subperiosteal suture placement.

There are currently three thoracotomy closure techniques: intracostal suture closure (whereby stitches perforate the rib); subperiosteal suture closure (whereby stitches are placed between the periosteum and the neurovascular bundle at the lower rib; Figure 1); and pericostal suture closure (whereby stitches are placed in the middle of the intercostal muscles, thus crushing the intercostal nerve against the rib).

Cerfolio et al.^(^ ^9^ ^)^ compared post-thoracotomy pain between patients undergoing pericostal suture closure and those undergoing intracostal suture closure. The authors reported that intracostal sutures resulted in significantly less postoperative pain. In another study, postoperative pain was evaluated in patients in whom the neurovascular bundle had been dissected before retractor placement in order to protect it from being compressed by the retractor.^(^ ^10^ ^)^ Many groups of authors have attempted to reproduce the results of the two aforementioned studies,^(^ ^11^ ^,^ ^12^ ^)^ having reached similar conclusions; some have attributed the reduction in pain to the protection provided by the muscle flap containing the bundle, whereas others have attributed it to the thoracotomy closure technique.

The present study was designed to compare, in a systematic manner, different techniques for neurovascular bundle preservation (NBP), in an attempt to determine their efficacy in reducing pain in the immediate postoperative period and during the in-hospital postoperative period.

## Methods

This was a prospective randomized clinical trial of candidates for elective thoracotomy in the Thoracic Surgery Department of the Federal University of São Paulo/Paulista School of Medicine, located in the city of São Paulo, Brazil. The trial was conducted between January of 2009 and January of 2010. All patients were blinded to the technique used. We initially selected 142 patients requiring thoracotomy. The inclusion criteria were being 18 years of age or older and agreeing to participate in the study. Most of the patients who were initially selected were excluded during the perioperative period on the basis of the following criteria: need for thoracoplasty or pleurectomy; occurrence of rib fractures caused by retractor placement; impossibility of performing epidural anesthesia; impossibility of using a visual analog scale in the postoperative period (because of prolonged intubation); and need for reintervention.

The study was approved by the Research Ethics Committee of the Federal University of São Paulo (Protocol no. 1323/09).

Before the surgical procedure, all patients underwent thoracic epidural anesthesia in a sitting position (puncture at the level of T5-T6), followed by local anesthetic infusion and epidural catheter placement. The incision was 8-10 cm in length. The latissimus dorsi was dissected and reflected posteriorly. The serratus anterior was identified, dissected, and reflected medially.

The intercostal muscles were separated midway between the upper and lower rib edges. A medium-sized Finochietto retractor with 4-cm blades was used in all procedures.

**Figure 2:**
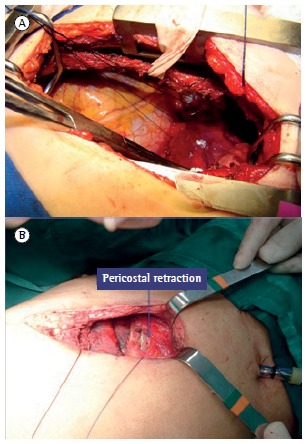
In A, intercostal muscle flap tied by a Penrose drain. In B, periosteal retraction.

The patients were randomized into two groups of 20 patients: the conventional thoracotomy group and the NBP group. In the group of patients undergoing conventional thoracotomy, the retractor was placed immediately after separation of the intercostal muscle. After the surgical procedure, intercostal space closure was achieved with pericostal sutures (absorbable polyglactin 1 suture material). In the NBP group, the intercostal muscle was dissected together with the neurovascular bundle, over a length of 5 cm, with the use of an osteotome and electrocautery, being therefore freed from the upper rib; the muscle flap was subsequently tied by a Penrose drain (Figure 2A) and retracted for the placement of the Finochietto retractor. After the surgical procedure, intercostal space closure was achieved with three stitches with subperiosteal sutures (a single layer of absorbable polyglactin 910 suture material; Figure 2B).

A visual analog scale ranging from zero to ten (i.e., from "no pain at all" to "the worst pain I have ever felt") was used for postoperative pain assessment. Patients used the visual analog scale to rate their pain on postoperative days 1, 3, and 5. All ratings were performed at 8:00 a.m. before any manual handling of patients, including bathing, physical therapy, and radiological examinations.

Objective postoperative pain assessment consisted of measuring the consumption of analgesics by patient demand (analgesics being prescribed and administered on the basis of self-reported pain).

Analgesia was achieved by the administration of 0.25% bupivacaine with 2 mg of morphine via an epidural catheter between 10:00 a.m. and 12:00 p.m. on postoperative days 1, 2, and, 3, the catheter being then removed. We analyzed the following parameters: duration of surgery; length of hospital stay; duration of chest tube drainage; pain intensity; consumption of analgesics by patient demand; and complications. Complications included segmental or lobar atelectasis, pneumonia, prolonged air leak (i.e., air leak for more than 3 days), and surgical wound complications, including seroma, hematoma, and infection.

Statistical analysis was performed with the chi-square test and the Student's t-test. Sample size was calculated by comparison with similar studies,^(^ ^11^ ^,^ ^13^ ^)^ the mean and standard deviation being taken into consideration; the expected response was 30%, with a value of p < 0.05. A descriptive analysis was performed with calculations of arithmetic means and standard deviations.

## Results

Of the 142 patients requiring elective thoracotomy during the study period, 40 (28%) were included in the study. Patients were excluded for the following reasons: lack of thoracic epidural anesthesia, in 38 patients; rib fracture during the intraoperative period, in 24; impossibility of assessing pain intensity (because of prolonged intubation), in 15; history of chronic analgesic use, in 12; chest wall invasion by lung cancer, in 8; and need for reintervention, in 5.

The 40 patients included in the present study were randomized into two groups of 20 patients: the conventional thoracotomy group and the NBP group. The mean age was 48.3 ± 14.5 years in the conventional thoracotomy group and 48.8 ± 17.3 years in the NBP group. The body mass index was 24.6 ± 3.7 kg/m^2^ in the former and 22.8 ± 4.5 kg/m^2^ in the latter. Of the 20 patients in the conventional thoracotomy group, 11 were male, and 7 were smokers. Of the 20 patients in the NBP group, 10 were male, and 8 were smokers. Table 1 shows the surgical procedures performed in each group of patients.

**Table 1:** Surgical procedures in the patients undergoing conventional thoracotomy (n = 20) and in those undergoing thoracotomy with neurovascular bundle preservation (n = 20).a

Surgical procedures	Groups	
	CT	NBP
Segmentectomy	7	9
Lobectomy	6	8
Mediastinal tumor resection	2	1
Metastasectomy	2	1
Diaphragmatic defect repair	1	
Pneumonectomy	1	1
Pleural tumor resection	1	

CT : conventional thoracotomy

NBP : neurovascular bundle preservation

a Values expressed as n of patients

We analyzed the duration of surgery, length of hospital stay, and duration of pleural drainage in the two groups of patients (Table 2). We found no significant differences between the two groups regarding any of the aforementioned parameters.

**Table 2:** Duration of surgery, length of hospital stay, and duration of chest tube drainage in the groups studied.a

Variables	Groups		p*
	CT	NBP	
Duration of surgery, min	206.00 ± 112.96	190.32 ± 86.08	0.53
Length of hospital stay, days	6.0 ± 5.3	6.3 ± 3.9	0.85
Duration of chest tube drainage, days	4.6 ± 2.7	4.3 ± 2.6	0.21

CT : conventional thoracotomy

NBP : neurovascular bundle preservation

a Values expressed as mean ± SD

* Student's t-test

Regarding the subjective assessment of postoperative pain, mean visual analog scale scores were higher in the conventional thoracotomy group than in the NBP group (p = 0.04). Although postoperative pain intensity on postoperative days 1, 3, and 5 was lower in the NBP group than in the conventional thoracotomy group, the difference was significant only on postoperative day 5 (p = 0.04; Table 3).

**Table 3:** Pain intensity, as assessed by visual analog scale scores, in the groups studied.a

Results	Groups		p*
	CT	NBP	
Highest score	6.14 ± 3.38	4.12 ± 2.63	0.04
Postoperative day 1	5.29 ± 3.94	3.58 ± 2.30	0.13
Postoperative day 3	2.86 ± 2.47	2.65 ± 1.83	0.51
Postoperative day 5	3.29 ± 2.36	1.50 ± 1.82	0.04

CT : conventional thoracotomy

NBP : eurovascular bundle preservation

a Values expressed as mean ± SD

* Student's t-test

Although the consumption of analgesics (tramadol hydrochloride and dipyrone) by patient demand was lower in the NBP group than in the conventional thoracotomy group, the difference was not significant. The mean consumption of tramadol hydrochloride was 1,025 ± 464 mg in the conventional thoracotomy group and 834 ± 568 mg in the NBP group (p = 0.22). The mean consumption of dipyrone was 16.67 ± 12.06 g in the former and 15.71 ± 11.73 g in the latter (p = 0.98).

Regarding the occurrence of postoperative complications, there were no significant differences between the conventional thoracotomy and NBP groups (28.18% vs. 30.77%; p = 0.58). In addition, none of the complications were attributable to the intervention (dissection of the neurovascular bundle and subperiosteal suture closure).

## Discussion

Although post-thoracotomy pain is a topic of great interest to thoracic surgeons, few studies have examined it. This might be due to the lack of objective data to quantify post-thoracotomy pain. In the present study, we sought to answer a simple question: what can surgeons do to minimize the pain of patients undergoing thoracotomy?

Although thoracic epidural anesthesia is still considered the gold standard for postoperative analgesia in thoracic surgery, it can cause nausea, vomiting, dizziness, and torpor (all of which are due to hypotension), as well as muscle weakness and urinary retention, in 15-20% of cases.^(^ ^14^ ^)^

Thoracic epidural anesthesia is contraindicated in patients with coagulation disorders and depends on the skill and experience of the anesthesiologist. Thirty-eight patients were excluded from the present study because of the impossibility of performing epidural anesthesia (either because it was medically contraindicated or because of technical difficulties). We do not question the benefits of thoracic epidural anesthesia, which is routinely used at our facility. However, it is sometimes impossible to use it.

In such cases, one alternative is intercostal nerve block under direct vision, covering one intercostal space above the incision and one below it, which can be beneficial within the first 24 h after surgery.

Muscle-sparing thoracotomy is a variant of posterolateral thoracotomy, which is an open procedure rather than a laparoscopic procedure. During muscle-sparing thoracotomy, the latissimus dorsi and serratus anterior muscle fibers are separated rather than sectioned. Although most pulmonary resections can be performed via a muscle-sparing thoracotomy, the efficacy of this approach in reducing postoperative pain remains controversial; some authors have suggested that it is ineffective in reducing postoperative pain,^(^ ^15^ ^)^ whereas others have reported that it significantly reduces postoperative pain.^(^ ^14^ ^)^ In our study, muscle-sparing thoracotomy was used in both groups, which were therefore not compared in terms of the technique.

The purpose of our study was to determine the extent to which the techniques that protect the neurovascular bundle from being compressed by the Finochietto retractor and the modified intercostal space closure technique can reduce postoperative pain.

Cerfolio et al.^(^ ^9^ ^)^ demonstrated the advantages of intracostal suture closure over conventional (pericostal) suture closure.

Few studies have examined the issue of intercostal nerve compression by the Finochietto retractor. Retractor-related factors contributing to the severity of post-thoracotomy pain include the amount of rib spreading, the size of the blades, and the area of contact between the retractor and the intercostal nerve.

In a study conducted in 2005, Cerfolio et al.^(^ ^10^ ^)^ proposed that an intercostal muscle flap containing the neurovascular bundle be harvested before placement of the retractor. The study included 114 patients, who were randomized to conventional thoracotomy or thoracotomy with intercostal muscle flap to protect the intercostal nerve. The authors found that the latter technique reduced postoperative pain.

The chest tube is known to play a role in postoperative pain, chest tube removal being often associated with a reduction in pain. In the present study, we found no significant difference between the two groups in terms of the duration of chest tube drainage. The results might have been different if we had.

In a prospective randomized study of 144 patients undergoing pulmonary resection, Wu et al.^(^ ^12^ ^)^ sought to determine whether the combination of intracostal suture closure with intercostal muscle flap provided better pain relief than did intracostal suture closure alone. Pain intensity was assessed by a visual analog scale in the period between postoperative day 1 and postoperative day 7, as well as in the period between postoperative week 2 and postoperative week 12. The combination of intracostal suture closure with intercostal muscle flap did not reduce postoperative pain when compared with intracostal suture closure alone.

In a prospective randomized study that involved 120 patients^(^ ^11^ ^)^ and that is similar to the present study, 60 patients underwent intercostal muscle flap and intracostal suture closure (for intercostal nerve protection) and 60 underwent conventional thoracotomy. Postoperative pain intensity was assessed by a visual analog scale and by analgesic consumption and was found to be lower at postoperative week 1 and at postoperative month 1 in the group of patients who underwent intercostal muscle flap and intracostal suture closure.

In the present study, the two groups of patients were found to be similar in terms of the length of hospital stay. This finding shows that the intervention reduces postoperative pain but not the length of hospital stay. This was not taken into consideration in studies similar to ours.^(^ ^10^ ^-^ ^12^ ^)^ We believe that this is due to the fact that postoperative pain is quite common and therefore a factor that is not relevant to discharge planning.

In the present study, the two groups were similar in terms of complication rates, and the intervention was ineffective in preventing the most common post-thoracotomy complications. Atelectasis and pneumonia are chief among the complications on which a reduction in pain might have any impact. In the present study, atelectasis occurred in 3 of the patients in the conventional thoracotomy group and in 2 of those in the NBP group. This difference might have reached statistical significance had our study involved a larger sample size. None of the complications observed in the present study were attributable to the intervention performed.

We conclude that the harvesting of an intercostal muscle flap before placement of the retractor and thoracotomy closure with subperiosteal sutures are measures that are not associated with additional morbidity or longer duration of surgery and can reduce in-hospital postoperative pain in patients undergoing thoracotomy.
