# Supplementary material for: The role of intercostal nerve preservation in acute pain control after thoracotomy
Source: J Bras Pneumol. 2014 Mar-Apr;40(2):164–70. doi: 10.1590/S1806-37132014000200010 (PMC4083640; doi:10.1590/S1806-37132014000200010)
Supplement: Translation

# Translation: O papel da preservação do nervo intercostal no controle da dor aguda pós-toracotomia^*^

**Authors:** Marco Aurélio Marchetti-Filho, Luiz Eduardo Villaça Leão, Altair da Silva Costa-Junior

**Affiliations:** Disciplina de Cirurgia Torácica, Universidade Federal de São Paulo/Escola Paulista de Medicina, São Paulo (SP) Brasil; Disciplina de Cirurgia Torácica, Universidade Federal de São Paulo/Escola Paulista de Medicina, São Paulo (SP) Brasil; Disciplina de Cirurgia Torácica, Universidade Federal de São Paulo/Escola Paulista de Medicina, São Paulo (SP) Brasil

**Keywords:** Dor pós-operatória, Analgesia, Toracotomia

## Abstract

**OBJETIVO:**

Avaliar se a dor aguda na recuperação intra-hospitalar devido a toracotomia pode ser efetivamente reduzida pelo uso de medidas intraoperatórias (dissecção do feixe neurovascular antes da colocação do afastador de Finochietto e preservação do nervo intercostal durante o fechamento).

**MÉTODOS:**

Foram selecionados 40 pacientes candidatos à toracotomia eletiva na Disciplina de Cirurgia Torácica, Universidade Federal de São Paulo/Escola Paulista de Medicina, em São Paulo (SP), os quais foram randomizados em dois grupos de 20 pacientes: grupo toracotomia convencional (TC) e grupo de preservação do feixe (PF) neurovascular. Todos os pacientes foram submetidos a anestesia peridural torácica e técnica de toracotomia poupadora da musculatura. A intensidade da dor foi determinada utilizando-se uma escala visual analógica no 1º, 3º e 5º dias pós-operatórios, assim como a medida do consumo de analgésicos por demanda do paciente.

**RESULTADOS:**

Houve uma diminuição significativa da intensidade da dor relatada somente no 5º dia pós-operatório no grupo PF quando comparado ao grupo TC (escore da escala analógica visual, 1,50 vs. 3,29; p = 0,04). Não houve diferenças significativas no consumo de analgésicos por demanda nos dois grupos.

**CONCLUSÕES:**

Em pacientes submetidos à toracotomia, a proteção do feixe neurovascular antes da colocação do afastador e a preservação do nervo intercostal no fechamento da toracotomia podem minimizar a dor no período intra-hospitalar.

## Introdução

A toracotomia pode ser considerada um dos procedimentos operatórios mais dolorosos da prática cirúrgica. A dor observada nos pacientes, tanto no pós-operatório imediato, quanto no tardio, se constitui em uma preocupação constante por parte dos cirurgiões torácicos, pois está amplamente demonstrado que pacientes com dor intensa pós-operatória têm um risco aumentado para o desenvolvimento de complicações, entre elas, as atelectasias e infecções pulmonares. ^(1,2) ^Por outro lado, a dor crônica é um motivo frequente do afastamento prolongado do trabalho, pois muitas vezes impede o paciente de exercer suas atividades habituais por meses após a operação. Muitos estudos apontam que a presença de dor intensa no pós-operatório imediato está relacionada a uma maior ocorrência de dor crônica.^(^ ^3^ ^-^ ^5^ ^)^

Para estudar a dor pós-operatória dependemos da percepção individual de cada paciente, o que dificulta a padronização dos estudos, seja pelo aspecto da subjetividade, seja pela dificuldade de mensurar sua intensidade.

A dor relacionada à estimulação dos receptores, chamada de dor nociceptiva (miofascial), pode estar relacionada a inúmeras situações que ocorrem nos diversos tempos da toracotomia (incisão cutânea; afastamento muscular; afastamento das costelas; trauma das junções costoesternais e costovertebrais; compressão do nervo intercostal; e lesão do parênquima pulmonar e da pleura parietal). A agressão ao nervo intercostal pode induzir à formação de um neuroma localizado que pode gerar estímulos persistentes, ocasionando os sintomas de hiperalgesia (definida como uma dor semelhante àquela sofrida por uma estimulação nociva) e alodinia (quando um estímulo tipicamente indolor é capaz de produzir dor). Nessa situação, a dor é denominada dor neuropática. A síndrome dolorosa pós-toracotomia pode ser então explicada por uma combinação de estímulos nociceptivos e neuropáticos.^(^ ^6^ ^,^ ^7^ ^)^

Atualmente, muitas operações torácicas são realizadas por videotoracoscopia; porém, ao analisarmos as ressecções pulmonares, entre elas, aquelas relacionadas ao tratamento do câncer de pulmão, mesmo com inúmeros estudos que dia a dia são publicados, enaltecendo as vantagens da videotoracoscopia, a realidade é diferente. Observa-se que, nos últimos anos, menos de 20% e de 10% das ressecções pulmonares maiores realizadas nos EUA e na Europa, respectivamente, são assistidas por vídeo, segundo dados da Associação Americana de Cirurgia Torácica.^(^ ^8^ ^)^

Em uma toracotomia convencional, quando não se adotam medidas para preservar o feixe neurovascular, potencialmente ocorre a lesão de dois nervos intercostais, um por ação do afastador de Finochietto e outro por compressão na sutura intercostal. Se partirmos do pressuposto que a dor pós-operatória está principalmente relacionada a lesões dos nervos intercostais, medidas que evitem essa compressão poderão minimizar esse sintoma.

Em relação às técnicas de fechamento da toracotomia, existem três possibilidades: sutura intracostal (os pontos perfuram a costela), sutura subperiostal (os pontos são aplicados entre o periósteo e o feixe neurovascular da costela inferior; Figura 1), e sutura pericostal (o ponto é aplicado no meio da musculatura intercostal, esmagando o nervo intercostal contra a costela).

**Figura 1:** Ilustração de sutura subperiostal.

Cerfolio et al.^(^ ^9^ ^)^ inicialmente avaliaram a dor pós-operatória quando se modificava a técnica de fechamento, comparando a sutura pericostal com a sutura intracostal. Os autores relataram que houve redução da dor quando se utilizava a sutura intracostal. Em outro estudo, foi avaliada a dor pós-operatória quando se dissecava o feixe neurovascular antes do posicionamento do afastador, protegendo-o da compressão do afastador.^(^ ^10^ ^)^ Os resultados desses estudos motivaram muitos grupos a reproduzi-los,^(^ ^11^ ^,^ ^12^ ^)^ chegando a conclusões semelhantes, alguns imputando a melhora à dissecção e proteção do retalho contendo o feixe, e outros imputando a melhora somente à técnica de fechamento da toracotomia.

O presente estudo foi desenhado de forma a comparar, de maneira sistemática, as técnicas de abordagem do feixe neurovascular, avaliando se realmente o uso de alguma técnica traz benefícios na minimização da dor no pós-operatório imediato e intra-hospitalar.

## Métodos

Foi realizado um ensaio clínico prospectivo e randomizado, no qual foram estudados todos os pacientes candidatos a toracotomia eletiva na Disciplina de Cirurgia Torácica da Universidade Federal de São Paulo/Escola Paulista de Medicina, em São Paulo (SP), no período entre janeiro de 2009 e janeiro de 2010. Todos os pacientes foram cegados em relação ao tipo de técnica utilizada. Inicialmente, foram selecionados 142 pacientes com indicação de toracotomia. Esses pacientes preencheram os seguintes critérios de inclusão: ter idade = 18 anos e concordar em participar do estudo. Os critérios de exclusão, aplicados durante o período perioperatório/operatório, foram responsáveis pela exclusão da maioria dos pacientes inicialmente selecionados: ser submetido a toracectomia ou pleurectomia; ocorrer fratura de costela na abertura do afastador, ser impossível realizar anestesia peridural, ser impossível aplicar a escala visual analógica no pós-operatório (devido a intubação prolongada) e haver necessidade de reintervenção.

O estudo foi submetido ao Comitê de Ética em Pesquisa da Universidade Federal de São Paulo, sendo aprovado sob o número 1323/09.

Em relação à técnica operatória utilizada, antes do início da operação, em posição sentada, os pacientes foram submetidos a anestesia peridural torácica (punção ao nível de T5-T6), seguida da infusão de anestésico local e posicionamento de cateter peridural. A incisão variou entre 8 e 10 cm de extensão, o músculo grande dorsal foi dissecado e rebatido posteriormente, assim como o músculo serrátil anterior foi identificado, dissecado e rebatido medialmente.

A musculatura intercostal foi aberta à meia distância entre os bordos inferior e superior das costelas. O afastador de Finochietto utilizado foi o de tamanho médio, com pá de 4 cm, sendo esse padrão seguido em todas as toracotomias.

Os pacientes foram randomizados para entrar em um dos dois grupos de estudo. Nos pacientes no grupo submetido à toracotomia convencional (TC), o afastador era posicionado imediatamente após a abertura do músculo intercostal. Após a realização da cirurgia, o espaço intercostal foi fechado com fio absorvível de poliglactina 1, por sutura simples, pela técnica pericostal. Nos pacientes do grupo com preservação do feixe neurovascular (PF), foi realizada a dissecção do músculo intercostal juntamente com o feixe neurovascular, numa extensão de 5 cm, liberando o músculo da costela superior, sendo utilizados rugina e eletrocautério; a seguir, o retalho muscular era laçado por dreno de Penrose (Figura 2A) e afastado para a colocação do afastador de Finochietto. Após a realização da operação, o espaço intercostal foi fechado por três pontos com o mesmo tipo de fio, também por sutura simples, mas utilizando-se a técnica subperiostal (Figura 2B).

**Figura 2:** Em A, retalho intercostal laçado por dreno de Penrose. Em B, descolamento do periósteo.

Para a avaliação da dor, foi utilizada uma escala visual analógica de zero a dez (variando de "sem dor" a "pior dor que já senti", respectivamente). Os pacientes classificaram a dor que estavam sentindo utilizando a escala analógica no 1º, 3º e 5º dias pós-operatórios. As avaliações foram padronizadas para ocorrer às 8 h da manhã, antes de qualquer manipulação do paciente, tais como banho, fisioterapia ou exames radiológicos.

Na avaliação objetiva, foi medido o consumo de analgésicos por demanda dos pacientes, os quais eram prescritos e somente administrados quando solicitados pelo paciente de acordo com a dor que estava sentindo.

A analgesia por cateter peridural foi feita pela administração de solução de bupivacaína a 0,25% associada a 2 mg de morfina, entre as 10-12 h da manhã, no 1º, 2º e 3º dias pós-operatórios, quando então o cateter era retirado. Foram estudados os seguintes parâmetros: tempo de cirurgia, tempo de internação, tempo de drenagem, escala de dor, quantidade de analgésico utilizada e complicações. Foram considerados como complicações os seguintes achados: atelectasia segmentar ou lobar, pneumonia, escape aéreo prolongado (superior a 3 dias) e complicações da ferida operatória (seroma, hematoma ou infecção).

A análise estatística foi realizada utilizando-se os testes do qui-quadrado e t de Student. O cálculo do tamanho da amostra foi feito através da comparação com estudos semelhantes,^(11,13) ^considerando sua média e desvio-padrão; a resposta esperada era de 30%, considerando-se um valor de p < 0,05. A análise descritiva foi realizada com as respectivas médias aritméticas e desvios-padrão.

## Resultados

No período de estudo, dos 142 pacientes com indicação de toracotomia eletiva, 40 (28%) foram incluídos no estudo. A exclusão dos pacientes ocorreu pela não realização de anestesia peridural torácica, em 38 pacientes; fratura de arco costal no período intraoperatório, em 24; impossibilidade de aplicação de escore de dor devido a intubação prolongada, em 15; história pregressa de uso crônico de analgésicos, em 12; invasão de parede torácica por tumor de pulmão, em 8; e necessidade de reintervenção, em 5.

Dos 40 pacientes incluídos no estudo, esses foram randomizados em dois grupos com 20 pacientes (grupos TC e PF). A média de idade dos pacientes dos grupos TC e PF foi de 48,3 ± 14,5 anos e 48,8 ± 17,3 anos, respectivamente, enquanto o índice de massa corpórea foi de 24,6 ± 3,7 kg/m^2^ e 22,8 ± 4,5 kg/m^2^. Nos grupos TC e PF, 11 e 10 pacientes eram do sexo masculino, respectivamente, enquanto 7 e 8 eram tabagistas ativos. A Tabela 1 demonstra os procedimentos cirúrgicos realizados nos pacientes por grupo de estudo.

**Tabela 1:** Procedimentos cirúrgicos realizados nos pacientes submetidos à toracotomia convencional (n = 20) ou à técnica de preservação do feixe neurovascular (n = 20).a

Procedimentos cirúrgicos	Grupos	
	TC	PF
Segmentectomia	7	9
Lobectomia	6	8
Ressecção de tumor no mediastino	2	1
Metastasectomia	2	1
Correção de defeito do diafragma	1	
Pneumonectomia	1	1
Ressecção de tumor pleural	1	

TC : toracotomia convencional

PF : preservação do feixe

a Valores expressos em n de pacientes.

Foram analisados o tempo de cirurgia, o tempo de internação e o tempo de drenagem pleural nos dois grupos (Tabela 2). Não houve diferenças estatisticamente significantes entre os dois grupos em relação a esses três parâmetros.

**Tabela 2:** Tempo de cirurgia, tempo de internação e tempo de drenagem nos grupos estudados.a

Variáveis	Grupos		p*
	TC	PF	
Tempo de cirurgia, min	206,00 ± 112,96	190,32 ± 86,08	0,53
Tempo de internação, dias	6,0 ± 5,3	6,3 ± 3,9	0,85
Tempo de drenagem, dias	4,6 ± 2,7	4,3 ± 2,6	0,21

TC : toracotomia convencional

PF : preservação do feixe

a Valores expressos em média ± dp

* Teste t de Student.

Em relação à avaliação da dor através da escala visual analógica, o grupo TC apresentou escores de dor maiores que o grupo PF, quando considerado o maior valor médio (p = 0,04). A avaliação da dor no 1º, 3º e 5º dias pós-operatórios mostrou valores menores no grupo PF, mas somente houve diferença significativa no 5º dia pós-operatório (p = 0,04; Tabela 3).

**Tabela 3:** Resultados da avaliação da intensidade da dor obtidos pelos escores da escala visual analógica nos grupos estudados.a

Resultados	Grupos		p*
	TC	PF	
Maior valor	6,14 ± 3,38	4,12 ± 2,63	0,04
1º dia pós-operatório	5,29 ± 3,94	3,58 ± 2,30	0,13
3º dia pós-operatório	2,86 ± 2,47	2,65 ± 1,83	0,51
5º dia pós-operatório	3,29 ± 2,36	1,50 ± 1,82	0,04

TC : toracotomia convencional

PF : preservação do feixe

a Valores expressos em média ± dp

* Teste t de Student

Em relação à avaliação da dor através da medida do consumo de analgésicos por demanda dos pacientes (cloridrato de tramadol ou dipirona sódica), apesar de o consumo desses ter sido menor nos pacientes do grupo PF, não houve diferenças significativas entre os dois grupos. A média de consumo de cloridrato de tramadol foi de 1.025 ± 464 mg e 834 ± 568 mg nos grupos TC e PF, respectivamente (p = 0.22), enquanto a de dipirona sódica foi de 16,67 ± 12,06 g e 15,71 ± 11,73 g (p = 0,98).

Em relação à ocorrência de complicações pós-operatórias, não houve diferenças significativas entre os grupos TC e PF (28,18% vs. 30,77%; p *= *0,58), não sendo observada nenhuma complicação que possa ser atribuída à intervenção (dissecção do feixe neurovascular e sutura subperiostal).

## Discussão

A ocorrência de dor pós-toracotomia é um assunto de relevante importância para o cirurgião torácico e, curiosamente, ainda é pouco estudada na literatura médica. Talvez a falta de dados objetivos para quantificá-la explique o pequeno número de estudos relacionados a essa ocorrência tão frequente. O objetivo do presente estudo foi o de tentar responder a uma pergunta simples: o que o cirurgião pode fazer para minimizar a dor de um paciente submetido a uma toracotomia?

A anestesia peridural torácica ainda é considerada o padrão ouro em analgesia pós-operatória em cirurgia torácica; porém, alguns inconvenientes podem ser atribuídos a ela, como a ocorrência de náuseas, vômitos, tontura e torpor (atribuídos a hipotensão), assim como fraqueza muscular e retenção urinária, alcançando índices que variam de 15-20% de ocorrência.^(^ ^14^ ^)^

A realização da anestesia peridural torácica possui algumas contraindicações principalmente relacionadas a distúrbios de coagulação e é dependente da experiência e destreza do anestesiologista. Em nosso estudo, 38 pacientes foram excluídos pela impossibilidade da realização da anestesia peridural, seja por contraindicação médica, seja por dificuldade técnica. Não temos dúvidas dos benefícios da anestesia peridural torácica e, rotineiramente, utilizamos esse tipo de anestesia em nosso serviço. Entretanto, somos privados de sua utilização com certa frequência.

Na impossibilidade de realização da anestesia peridural torácica, uma alternativa é a realização da anestesia do nervo intercostal sob visão direta, abrangendo um espaço intercostal acima e um abaixo da incisão, que pode trazer benefícios nas primeiras 24 h de pós-operatório.

Em relação à técnica operatória, nas cirurgias torácicas ditas abertas, isto é, quando não se usa o aparato da videotoracoscopia, uma variante da técnica da toracotomia posterolateral é a técnica de preservação muscular. Essa abordagem, na qual os músculos grande dorsal e serrátil anterior têm suas fibras afastadas e não seccionadas, permite a realização da grande maioria das ressecções pulmonares, mas seu impacto em termos de minimização da dor ainda é controverso; alguns autores apontam sua ineficácia na diminuição da dor pós-operatória,^(^ ^15^ ^)^ enquanto outros relatam um impacto significativo na sua redução.^(^ ^14^ ^)^ Em nosso estudo, a técnica poupadora da musculatura foi aplicada nos dois grupos, não sendo, portanto, um fator de comparação.

O propósito de nosso estudo foi avaliar em que proporção as técnicas que preservam o feixe neurovascular antes de esse ser comprimido pelo afastador de Finochietto, assim como a modificação no tipo de fechamento do espaço intercostal, podem influenciar na dor pós-operatória.

Em relação à técnica de fechamento, Cerfolio et al.^(^ ^9^ ^)^ demonstraram as vantagens da técnica intracostal em relação ao fechamento convencional (pericostal).

Quando analisamos a compressão do nervo intercostal pelo afastador, não existem muitos estudos que avaliem isoladamente essa questão, e possíveis variáveis podem contribuir para a intensidade da dor, como o tamanho da abertura do afastador, o tamanho da pá do afastador e a consequente maior ou menor superfície de contato com o nervo intercostal, entre outras.

Em 2005, Cerfolio et al.^(^ ^10^ ^)^ propuseram a dissecção do retalho intercostal contendo o feixe neurovascular antes da colocação do afastador. Seu estudo incluiu 114 pacientes randomizados para toracotomia convencional ou para dissecção de retalho muscular e, quando a dor pós-operatória foi analisada, os resultados mostraram um amplo benefício da técnica de proteção do nervo intercostal.

Quando nos propomos a avaliar a dor pós-operatória, é fato que o dreno de tórax exerce influência, pois é observação frequente o relato de diminuição da dor após a retirada do dreno. Em nosso estudo, não observamos uma significância estatística entre os dois grupos em relação ao tempo de permanência do dreno de tórax, fato que poderia comprometer nossos resultados.

Wu et al.,^(^ ^12^ ^)^ em um estudo prospectivo com 144 pacientes randomizados, compararam a técnica do retalho de músculo intercostal associada à técnica de fechamento intracostal com a técnica de fechamento intracostal isoladamente. A intensidade da dor foi medida do 1º ao 7º dia pós-operatório e de 2 a 12 semanas do pós-operatório através de uma escala visual analógica. A dissecção do retalho não foi eficiente em reduzir a dor pós-operatória quando comparada ao grupo do fechamento intracostal isoladamente.

Em um estudo prospectivo e randomizado^(^ ^11^ ^)^ semelhante ao nosso com 120 pacientes, 60 foram submetidos a dissecção de retalho intercostal e fechamento intracostal, e 60 foram operados pela técnica convencional. Os resultados obtidos demonstraram uma redução da dor, avaliada por escala visual analógica e consumo de analgésicos, na 1ª semana e no 1º mês pós-operatórios nos pacientes submetidos a medidas de proteção do nervo intercostal.

Quando avaliamos o tempo de internação hospitalar, obtivemos resultados semelhantes nos dois grupos, mostrando que a intervenção, apesar de reduzir a dor, não diminui o tempo de internação. Esse dado não foi considerado em estudos semelhantes ao nosso,^(10-12) ^pois acreditamos que a queixa de dor, por ser muito frequente no pós-operatório, não se constitui em um fator relevante na alta hospitalar.

Os índices de complicações também foram semelhantes nos dois grupos, e a intervenção não se mostrou eficaz na prevenção das complicações mais comuns no pós-operatório da toracotomia. Consideramos que, entre as complicações que possivelmente poderiam ser influenciadas pela a diminuição da dor, a atelectasia e a pneumonia seriam as mais importantes. No nosso estudo, observamos 3 e 2 pacientes com atelectasia nos grupos TC e PF, respectivamente; talvez em um estudo com um número maior de pacientes, esses achados possam atingir significância estatística. Não observamos nenhuma complicação que possa ser atribuída à intervenção realizada.

Concluímos que a dissecção do retalho de músculo intercostal antes da colocação do afastador, associado à técnica de fechamento da toracotomia por sutura subperiostal, não agrega morbidade, bem como não aumenta o tempo operatório, e possibilita a redução da dor no período intra-hospitalar.
